# Investigations on Potential Applications of CaMg(CO_3_)_2_ Nanoparticles

**DOI:** 10.3390/molecules28010316

**Published:** 2022-12-30

**Authors:** Ihn Han, Chulwoo Rhee, Doyoung Kim

**Affiliations:** 1 Plasma Bioscience Research Center, Kwangwoon University, Seoul 01897, Republic of Korea; 2 Department of Electrical and Biological Physics, Kwangwoon University, Seoul 01897, Republic of Korea; 3 Department of Earth and Environmental Sciences, Chungbuk National University, Seoul 28644, Republic of Korea

**Keywords:** calcium magnesium carbonate nanoparticles, dolomite, limestone, nanoparticle agriculture, calcium absorption

## Abstract

Calcium magnesium carbonate nanoparticles (CaMg(CO_3_)_2_ NPs), well-known as dolomite, are formed by the replacement of half of the calcite minerals of limestone. The dolomite (CaMg(CO_3_)_2_) nanoparticles are composed of calcite (CaCO_3_) and magnesium carbonate (MgCO_3_), both of which offer promising strategies for maintaining growth and development in mammals and agricultural plants. A grounded mixture of dolomite limestone was prepared via colloidal precipitates for the synthesis of CaMg(CO_3_)_2_ NPs, and their characteristics were examined using XRD, particle size analysis by DLS, and surface morphology by SEM and TEM. X-ray photoelectron spectroscopy was used to investigate the binding energy of each element of the dolomite NPs. Spectroscopy revealed that colloidal precipitation is the ideal method for producing NPs. We assessed the numerous beneficial impacts of CaMg(CO_3_)_2_ NPs in diverse sectors such as agriculture, cancer treatment, and microbiology in this study. Furthermore, an in vivo study was also carried out on chickens to observe the effects of CaMg(CO_3_)_2_ NPs. The obtained results showed that the treated group with CaMg(CO_3_)_2_ NPs maintained a more uniform calcium absorption rate than the control group did. The findings of this study suggest that CaMg(CO_3_)_2_ NPs operate as a stimulant for plants and as an inhibitory agent for bacteria and cancer cells.

## 1. Introduction

Calcium carbonate minerals such as calcite and aragonite are component of pearls, eggshells, and shellfish skeletons and form limestone, a sedimentary rock [1,2]. Calcium carbonate plays an important role in the agricultural industry via agricultural lime and can be found in limescale, produced by combining carbonate ions and calcium ions in hard water. It is used in medicine as a calcium supplement or an antacid, but too much of it can cause ailments, such as hypercalcemia and problems in the digestive system [3,4]. Calcium carbonate is also widely used in the construction sector as a building material [5,6]. In a blast furnace, calcium carbonate is also used to purify iron from iron ore. The carbonate is calcined in situ to produce calcium oxide, which solidifies as a slag containing different impurities and separates from the refined iron [7,8,9,10,11].

Magnesium is a vital mineral in the human body, as it is involved in over 300 enzyme processes. It has a variety of activities, including assistance in muscle and neuron function, blood pressure regulation, and immune system support [12,13]. Magnesium is required for a variety of biological activities. Sufficient intake of the mineral can aid in the prevention or treatment of chronic illnesses such as Alzheimer’s disease, type 2 diabetes, cardiovascular disease, and migraine [14,15].

A single dose of calcium of no more than 500 mg should be given for optimum absorption. Magnesium helps deposit calcium into our bones by suppressing parathyroid hormone and stimulating calcitonin, thereby helping to prevent osteoporosis [16]. However, calcium can be poisonous without magnesium, accumulating in soft tissue and causing arthritis. A calcium-to-magnesium ratio of 2:1 is a decent rule of thumb [17]. While the majority of studies in academia have focused on the function of calcium in bone health, magnesium is also necessary for bone formation [18,19]. Magnesium may benefit bone health both directly and indirectly by assisting in the regulation of calcium and vitamin D levels, two additional essential minerals for bone health [20].

In this study, we synthesized dolomite nanoparticles (CaMg(CO_3_)_2_ NPs) and evaluated potential applications as an effective source of both calcium and magnesium. Nanoparticles of CaMg(CO_3_)_2_ would enhance organism absorption of Ca and Mg. In addition, dolomite NPs offer an array of industrial applications owing to their non-toxicity, biocompatibility with bodily fluids, high porosity, and high surface-area-to-volume ratio [21,22]. Because of such wide applications, our goal is to increase calcium deposition rate by using synthetic NPs. Following the synthesis of dolomite NPs, we investigated numerous impacts in agriculture, cancer treatment, and antibacterial properties. Additionally, we carried out an in vivo study on chickens to observe the beneficial effects of CaMg(CO_3_)_2_. We found that NPs have a variety of roles in several fields, such as an inhibitory agent and stimulator of several plant growth hormones. The CaMg(CO_3_)_2_ NPs were discovered to have possible positive effects that are not hazardous to human health and may boost absorption of calcium and magnesium in the human body in the future.

## 2. Results and Discussion

### 2.1. Effect of CaMg(CO_3_)_2_ on Rice Seed Germination

The effect of dolomite NPs on plant growth was investigated in this work. The germination rate of rice seeds (*Oryza sativa* L. ‘Hopyeong’) treated with CaMg(CO_3_)_2_ NPs was assessed, and results were contrasted with the control. Rice seeds were used to determine the germination rate after dolomite NP treatment, and results were compared with a control group. Seeds were divided into two groups, control and treated with 1% CaMg(CO_3_)_2_ NPs. They were then placed in a plant chamber with a humidity level of 60% and a temperature of 25 °C to promote germination in a plant culture dish. Various time intervals up to 25 days were used to observe the seed germination rate. We chose 15 rice seeds to compare the germination rates between the control and 1%-CaMg(CO_3_)_2_-NP-treated groups, and we used this approach more than three times. The 15 rice seeds were selected to observe the germination rate in the control group and the group treated with dolomite NPs. The germination rates in control and treated groups were observed after 9 days.

**Figure 1:**
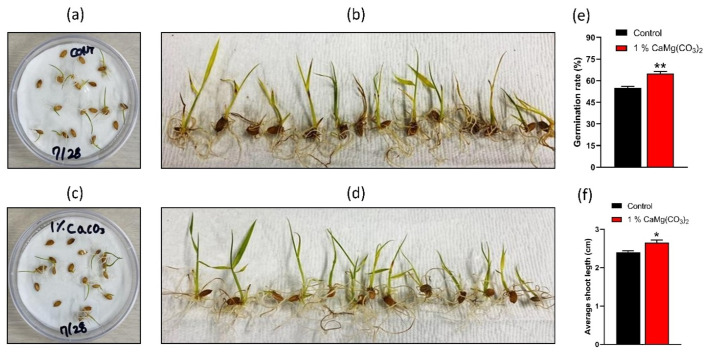
The application of CaMg(CO_3_)_2_ in agriculture. (**a**,**b**) Photographs of the seed germination and rice plant at day 1, 9, 17, and 25 in a control group and a CaCO_3_-treated group, respectively. (**c**,**d**) Photographs after harvesting to measure shoot length in both control and treated groups. The quantitative analyses of rate of germination and average shoot length were given in (**e**,**f**), respectively. The significance was calculated using Microsoft Excel (MS Office 365). * *p* < 0.05 and ** *p* < 0.01 indicate differences between treatment groups.

The seed germination test determines the percentage of seeds that are alive and possess the ability to grow under specific conditions. Our result shows that various NPs positively affect plant growth [23,24,25,26]. In this work, we have observed the effect of CaMg(CO_3_)_2_ NPs on the seed germination and shoot length of the rice plant. Figure 1 shows the results obtained during the experiment. Figure 1a,b shows the photograph of germinated seed and plant shoots in the control group at day 1, 9, 17, and 25, respectively. Similarly, Figure 1c,d shows harvested rice plants on day 25, in the control group and 1%-CaMg(CO_3_)_2_-NP-treated group, respectively. Figure 1e,f indicates the quantitative analysis of the germination rate and average shoot length, respectively. The CaMg(CO_3_)_2_ NPs are found to have a significant effect on seed germination: germination rate is 50% in the control group at day 17, while it is 65% in the 1%-CaMg(CO_3_)_2_-NP-treated group. The average shoot length of the rice plant is slightly higher in the 1%-CaMg(CO_3_)_2_-NP-treated group compared to the control group. The average shoot length in the control group was 2.4 cm, while in the 1%-CaMg(CO_3_)_2_-NP-treated group, it is 2.6 cm. By taking the 1% quantity of the CaMg(CO_3_)_2_ NPs into account, we can observe the advantageous effect on seed germination; in the CaMg(CO_3_)_2_-NP-treated group, it is 2.6 cm. By taking the 1% quantity of the CaMg(CO_3_)_2_ NPs into account, we can observe the advantageous effect on seed germination.

### 2.2. Antibacterial Activity of the CaMg(CO_3_)_2_

For the antibacterial activity test using dolomite NPs, the bacteria Gram-negative *E. coli* (ATCC25922) was selected. The microorganism *E. coli* was first cultured in LB medium for 8 to 24 h at 37 °C in a shaking incubator. After 24 h of incubation, the cultured *E. coli* was divided into two different experimental groups, a control group and a group treated with dolomite NPs. After treatment with different dilutions ranging from 10^1^ to 10^3^ NPs, treated and control groups were spread on the nutrient agar plate and incubated at 37 °C for 24 h. The antibacterial activity was observed on nutrient agar plate on both groups post-incubation.

**Figure 2:**
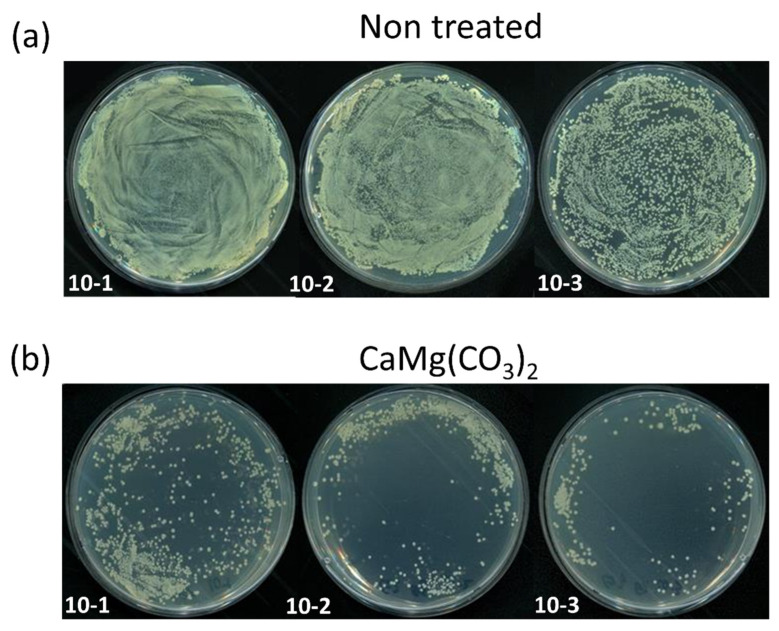
The antibacterial activity of the CaMg(CO_3_)_2_ on *E. coli*. (**a**) Control group, (**b**) CaMg(CO_3_)_2_-treated group.

**Figure 3:**
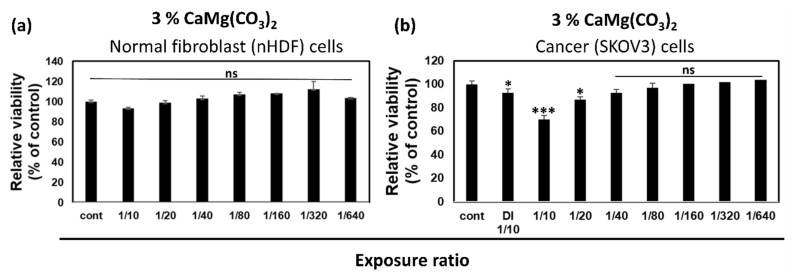
The cytotoxicity effects of 3% CaMg(CO_3_)_2_ on (**a**) normal fibroblast nHDF cell lines and on (**b**) cancer SKOV3 cell lines. Microsoft Excel was used to compute the significance (MS Office 365). Differences between treatment groups are shown by * *p* < 0.05, and *** *p* < 0.001.

Antibacterial activity was demonstrated using Gram-negative *E. coli* bacteria at various dilutions. Figure 2a,b shows the control group and the treated group, respectively, at three different dilutions. The growth rate of bacteria was greatly inhibited in all of the dilutions tested when the bacteria were treated with CaMg(CO_3_)_2_. According to these findings, CaMg(CO_3_)_2_ appears to be a promising choice for bacterial inhibition. The obtained results in Figure 3 show that, following CaMg(CO_3_)_2_ NP treatment at various dilutions, the treated groups were dramatically influenced. 

### 2.3. Cytotoxicity of the CaMg(CO_3_)_2_

The cytotoxic effect of dolomite NPs was also observed in this study. For that, two cell lines, normal fibroblast (nHDF) and ovarian cancer (SKOV3), were selected. The cells were treated with different dilutions, 3% of dolomite NPs. SKOV3 ovarian cancer cells were obtained from the American Type Culture Collection (ATCC, Rockville, MD, USA). The cells were cultured in a DMEM (HyClone, Logan, UT, USA) which contained 10% (*v*/*v*) heat inactivated fetal bovine serum (FBS) (Gibco, Thermo Fisher Scientific, Waltham, MA, USA) and 1% (*v*/*v*) antibiotics (Gibco, Thermo Fisher Scientific, Waltham, MA, USA) until an adequate cell number was obtained. Throughout the experiment, the cells used were less than passage, 8 cells seeded in 96-well plates with density 1 × 10^4^ cells/mL in 100 μL DMEM media. The culture was maintained at 37 °C in 5% CO_2_ and 95% humidity. In our study, we use the serum-free media for CaMg(CO_3_)_2_ NPs that had been separated from the calcium carbonate; fine particles were freely precipitated off of them, increasing the concentration of calcium carbonate solids to 12.0 to 20.0% by weight. In our investigation, we first prepare a stock solution that has CaMg(CO_3_)_2_ nanoparticles at a 3% concentration. Then, we performed numerous dilutions varying from 1:10 to 1:640 to assess the results of the cell cytotoxicity assay.

The cytotoxic effects of CaMg(CO_3_)_2_ nanoparticles were also observed on normal fibroblast (nHDF) and cancer SKOV3 cell lines. The results are shown in Figure 3. Figure 3a shows the effect of CaMg(CO_3_)_2_ on normal nHDF, and Figure 3b indicates the cancer SKOV3 cells. On the specified dilutions, it was observed that CaMg(CO_3_)_2_ had no effect on normal nHDF. On the other hand, Cancer SKOV3 cells were greatly suppressed at a 1:10 exposure ratio after employing 3% CaMg(CO_3_)_2_.

### 2.4. Changes in Appearance and Quality of Eggs with CaMg(CO_3_)_2_ NPs: In Vivo Experiment

The same amount of oyster shells and limestone in the existing radiation scattering system was replaced with dolomite nanoparticles, and the effect on spawning was observed and recorded. Self-feed containing dolomite nanoparticles was fed in a certain amount and for a certain period of time. The state of the eggshell from the egg was observed 3 days after feeding and 3 days after stopping feeding. In both cases, the thickness of the eggshell and height of the yolk layer was measured and recorded in the Haugh Unit of the yolk. Thus, the obtained results were compared with the control and the treated groups.

For up to 18 days, the changes in eggshell appearance were tracked. In the first seven days, there was not much external change. When one ingredient in the feed is changed, it can be challenging to detect the difference within the first seven days. On the eighth day, the color faded a little, and the luster seemed to be lost, but on the ninth and tenth days, both had completely recovered. Interestingly, when dolomite nanoparticle feeding stopped on the 13th–15th days, a significant change in egg quality was observed, and on the 18th day, bumps appeared on the surface of the shell, and the strength of the shell was relatively weak.

**Figure 4:**
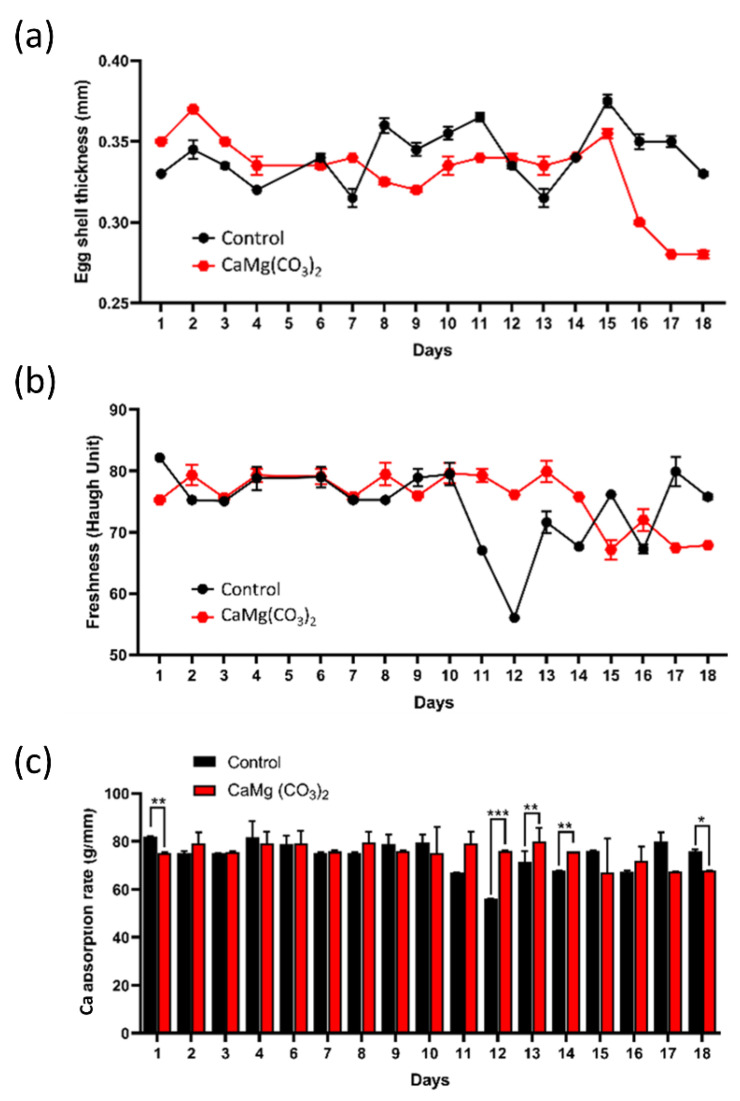
(**a**) The eggshell thickness with respect to the days in control and treated groups. (**b**) The egg freshness measured in the control and experimental group, up to 18 days. (**c**) The calcium absorption rate in control and CaMg(CO_3_)_2_-treated groups. The significance was calculated using Microsoft Excel (MS Office 365). * *p* < 0.05, ** *p* < 0.01, and *** *p* < 0.001 indicate differences between treatment groups.

A Bluetec digital caliper with a minimum scale of 0.01 mm was used to gauge egg thickness. For quality control and research purposes, this device uses ultrasound to measure the thickness of shells without breaking them. A Digital Haugh Tester (Lakeview Dr, Bountiful, UT, USA), an instrument which measures the height of egg albumen and allows for the manual determination of the Haugh Unit (HU) quickly and accurately, was also used to determine freshness of the eggs. The eggshell thickness changed as the days passed, as indicated in Figure 4b. Eggs laid by the chickens fed with dolomite nanoparticles also formed well in the third layer, and in some cases, the highest record of HU was observed (Figure 4b). The value of the treated group showed significant change, unlike the test group, which maintained a constant freshness, as inferred from the Haugh Unit. Consequently, it was found that while the freshness of the egg improved, the eggshell got thinner over time in treated groups. Taken all together, the treated group with CaMg(CO_3_)_2_ NPs maintained a more uniform calcium absorption rate compared to the control group, as shown in Figure 4c.

## 3. Materials and Methods 

### 3.1. Production Mechanism of Dolomite Nanoparticles

**Figure 5:**
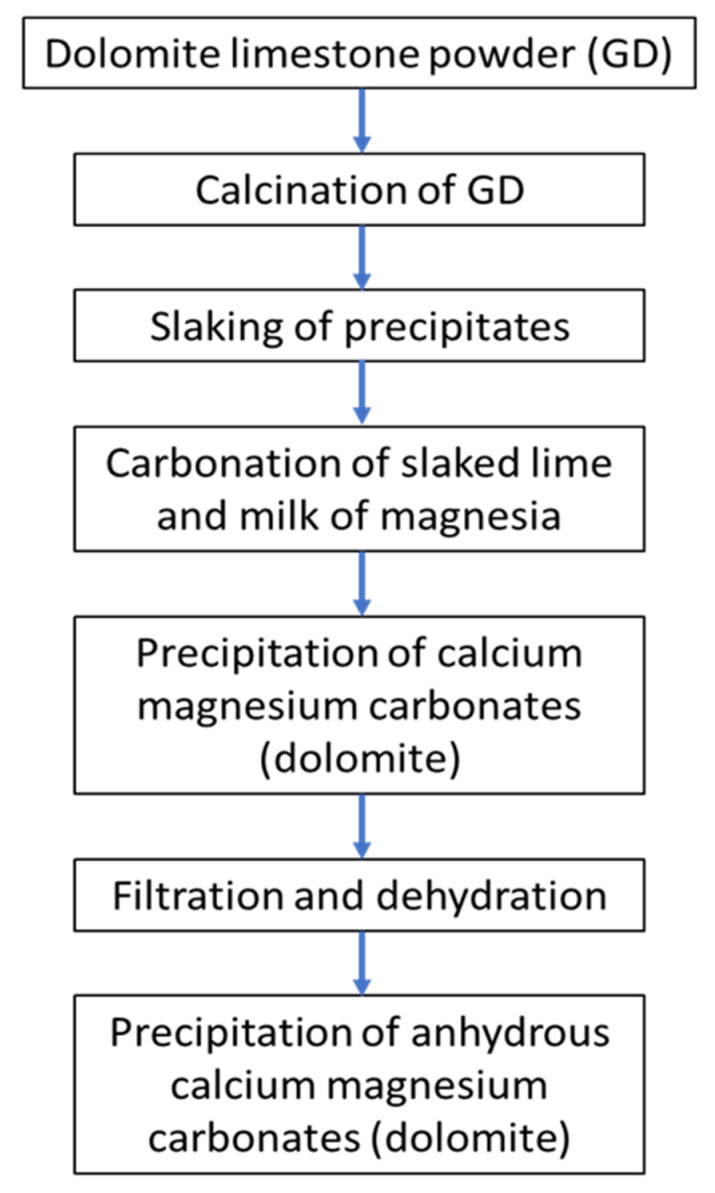
Production mechanism of dolomite nanoparticles from dolomite limestone by colloidal precipitation method.

In this study, we produced dolomite NPs using a colloidal precipitate method and dolomite limestone (dolostone, a kind of carbonate sedimentary rock) (Daesung GMTech, Gangwondo, Republic of Korea) as raw materials; flow chart representation is given in Figure 5. The production mechanism of dolomite nanoparticles is as follows: (1) Dolomite limestone (CaMg(CO_3_)_2_) fragments are ground into dolomite particles (GD) of 30–50 mm in diameter; (2) After homogenizing ground (GD) precipitate, the GD is calcinated in a lime kiln (rotary type, vertical type, kiln such as BK, Mertz, etc.) at 1000 °C ± 100 °C to make a mixture of quicklime (CaO) and magnesium oxide (MgO) with the evaporation of carbon dioxide (CO_2_).

(1) $$\mathrm{CaMg}{\left({\mathrm{CO}}_{3}\right)}_{2}\overset{\mathrm{Heat}}{\to }\mathrm{CaO},\;\mathrm{MgO}+2{\mathrm{CO}}_{2}↑$$

The mixture of quicklime and magnesium oxide is mixed with water (H_2_O) to produce lime milk (Ca(OH)_2_).

(2) $$\mathrm{CaO},\;\mathrm{MgO}+2{\;H}_{2}O\;\to \;\mathrm{Ca}{\left(\mathrm{OH}\right)}_{2}↓+\;\mathrm{MgO}+H_{2}O$$

The lime milk should be separated and removed from the magnesium oxide (MgO) (Daesung GMTech, Republic of Korea) and water. The remaining mixture of magnesium oxide and water is charged into an autoclave (ST-105G, JEIO Tech Co., Ltd., Daejeon, Republic of Korea) and hydrated at steam pressures in the range of 1.7 to 7 atmospheres and temperatures of 115 to 165 °C to produce magnesium hydroxide, Mg(OH)_2._

(3) $$\mathrm{MgO}+H_{2}O\;\left(\mathrm{within}\;\mathrm{autoclave}\right)\;\to \;\mathrm{Mg}{\left(\mathrm{OH}\right)}_{2}$$

The lime milk (Ca(OH)_2_) and magnesium hydroxide (Mg(OH)_2_) are mixed together, and then the mixture is carbonated through the following reaction by adding carbon dioxide.

(4) $$\mathrm{Ca}{\left(\mathrm{OH}\right)}_{2}+\;\mathrm{Mg}{\left(\mathrm{OH}\right)}_{2}+2{\mathrm{CO}}_{2}\to \;\mathrm{CaMg}{\left({\mathrm{CO}}_{3}\right)}_{2}↓+2H_{2}O\;$$

The lime milk and magnesium hydroxide are aged for three to seven days. The concentration is adjusted (4% to 30%) as well as the temperature (0 °C to 30 °C), depending on the target size and shape of the precipitated dolomite particles. Natural aging for 5–7 days converts the lime milk and magnesium hydroxide into colloidal dolomite nanoparticles of 0.04 µmØ, which grows up to 0.06 µmØ and 0.08 µmØ particle size through the carbonate reaction (d). Filter presses (CFS-50; Sinoo Mechanics Co., Gyunggido, Republic of Korea) and decanter centrifuges (HANIL SCI-MED, Daejeon, Republic of Korea) are used to dehydrate the colloidal dolomite. The dolomite particles are then dehydrated. The dolomite NP precipitates are then gathered during the drying process.

### 3.2. Characterization of Dolomite Nanoparticles

Characterization of dolomite nanoparticles was done by XRD (SmartLab, Rigaku, Japan), XPS (K-Alpha+, Thermo Fisher Scientific, Waltham, MA, USA), SEM (SU8010, Hitachi, Japan), and TEM (LIBRA 120, Carl Zeiss, Germany). XRD patterns were captured on a PANanalytical X’pert PRO (ID-OEM-1, Malvern Panalytical, Morgan Hill, CA, USA) between 10–75° (2), with a step size of 0.02°, and matched with an ICDD card using X’pert High Score software (Malvern Panalytical, Morgan Hill, CA, USA) to confirm phase formation. On the JEOL 2100 F (JEM 2100F, JEOL, Austin, TX, USA), morphological features were also recorded (200 kV; transmission electron microscope). The nanoparticle of dolomite hydrodynamic sizes was measured by dynamic light scattering using an ELSZ-1000 (Otsuka Electronics Co., Ltd., Osaka, Japan). The detailed content of nanoparticles was analyzed by the ICP-MS (Agilent 7900 ICP-MS), Agilent Technologies, Santa Clara, CA, USA) and DLS (Zetasizer nano ZS, Malvern, UK).

#### 3.2.1. The Crystallinity XRD and DLS Study on Dolomite NPs

**Figure 6:**
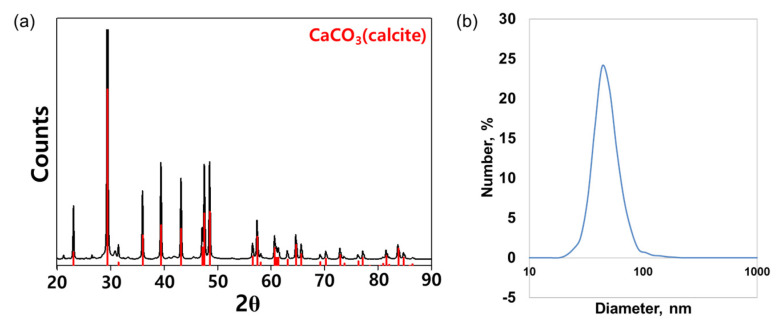
XRD and DLS analyses of CaMg(CO_3_)_2_. Notes: (**a**) XRD pattern of the purified CaMg(CO_3_)_2_. The Braggs reflections were identified in the XRD. (**b**) The distribution of hydrodynamic diameter of CaMg(CO_3_)_2_ measured by DLS. Abbreviations: XRD, X-ray diffraction; DLS, dynamic light scattering; CaMg(CO_3_)_2_, dolomite nanoparticles.

The morphological nanoparticle was analyzed by using X-ray diffraction spectra (XRD), and particle size distribution was examined by dynamic light scattering (DLS) (Malvern Panalytical, Morgan Hill, CA, USA). As dolomite is the double carbonate of calcium and magnesium, it consists of calcium carbonate and magnesium carbonate intercalating each other. Dolomite has the same hexagonal crystal system as calcite. According to XRD patterns, dolomite nanoparticles have a calcite structure (Joint Committee on Powder Diffraction Standards [JCPDS] No 47-1743) and exhibit high crystallinities (Figure 6). The overall XRD pattern showed sharper and intense peaks of 30, which was attributed to the larger particle size of the nanoparticle. Scherrer’s equation was used to calculate the crystallite sizes from the XRD peaks [27]. The results were 0.04 and 0.08 m, respectively. The XRD peaks of dolomite nanoparticle sample concentrated at 2Ø values of 22.0°, 30.0°, 35°, 40°, 45°, 50°, 60°, and 65° showed that the major mineral in the collected nanoparticle sample is calcite (PDF#86-0174). Calcium carbonate has three polymorphic crystalline forms: calcite, vaterite, and aragonite. Calcite is the most thermodynamically stable among the three [28,29]. During nanoparticle formation, the average crystalline size was measured to be around 1.0~12.0 µm Ø by the Debye–Scherrer formula.

Dynamic light scattering (DLS) was used to assess the mean particle size and size distribution of dolomite nanoparticles in kerosene using a ZEN3600 Zetasizer (Malvern Instruments Ltd.). The experiment was conducted at a temperature of 25 °C. Utilizing Mg–C nanoparticles produced in kerosene after ultrasonic treatment for 5 min, the sample measurement was done five times, with the average result being recorded. The sharp peak of this sample shows that the average particle size of 91.3% of the sample is 1.05 nm [30].

#### 3.2.2. Nanoparticle Surface SEM and TEM Study

**Figure 7:**
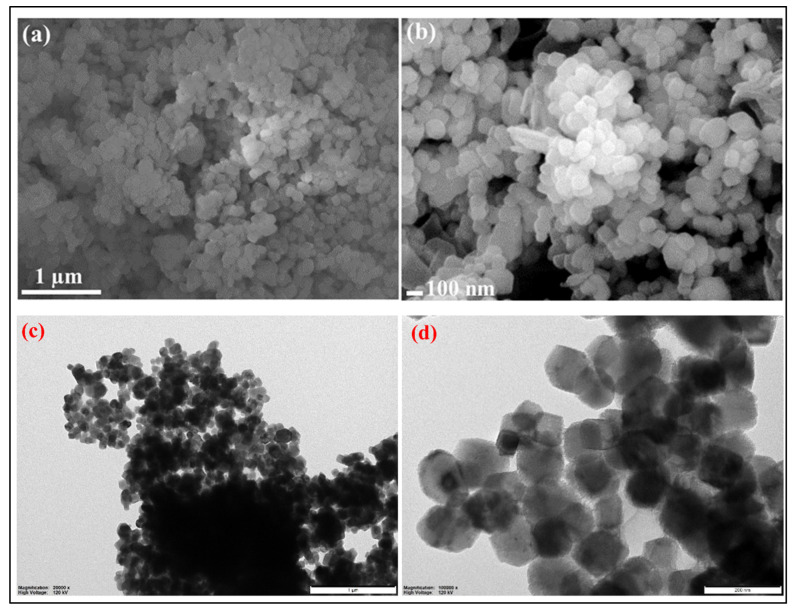
SEM-FES and TEM analyses of CaMg(CO_3_)_2_. Notes: (**a**,**b**) SEM-FES images of the purified CaMg(CO_3_)_2_ at 1 µm and 100 nm, (**c**,**d**) TEM images of CaMg(CO_3_)_2_ showing the particles’ shapes at the selected area diffraction pattern (SAED) of CaMg(CO_3_)_2_ with respective SAED apertures. The scale bars are 1 µm and 200 nm, respectively. Abbreviations: SEM for scanning electron microscopy; TEM for transmission electron microscopy; CaMg(CO_3_)_2_ for dolomite nanoparticles.

The surface morphological study of dolomite nanomaterials in different magnifications is illustrated in Figure 7a,b by using field emission scanning electron microscopy. The SEM results show that nanoparticles have a rectangular or cube shape, with size ranging from 100–150 nm. The dolomite nanoparticle size distribution is analyzed by TEM (transmission electron microscope) at different magnifications for highlighting the distinct features of the nanoparticles. TEM micrograph results confirmed that the dolomite nanoparticle has rhombohedral and hexagonal form. The TEM analysis shows various sizes of nanoparticles which are 1 µm and 200 nm at 20,000× and 10,000×, respectively [30,31] (Figure 7c,d).

#### 3.2.3. The Surface Chemical Analysis Using XPS

**Figure 8:**
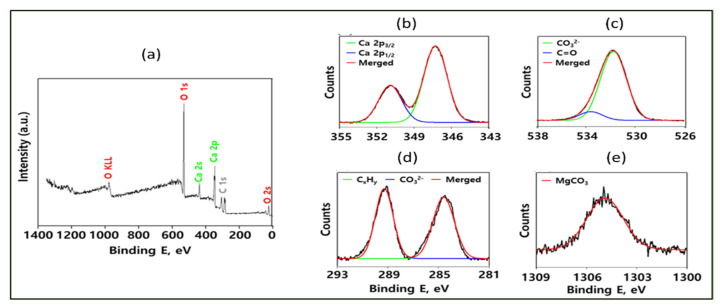
(**a**) XPS spectra of different elements of CaMg(CO_3_)_2_, dolomite nanoparticles. (**b**) Calcium’s 2p3/2 and 2p1/2 core levels spectra of nanoparticles. (**c**) CO_3_^2−^ and C=O levels spectra of thick film. (**d**) CxHy and CO_3_^2−^. (**e**) MgCO_3_ in binding energy. (**e**) Survey spectra of the prepared sample. Abbreviations: XPS for X-ray Photoelectron Spectroscopy.

In this XPS (K-Alpha+, Thermo Fisher Scientific, Waltham, MA, USA) analysis, we investigated the chemical characteristics and binding states of the nanoparticle using three different CaP films to measure the binding energy of the nanoparticles. The survey spectra confirmed the presence of calcium, magnesium, oxygen, and carbon in their binding energy (BE) values. PS analysis has more vivid results for nanoparticle samples and explains the binding energy of Ca, CO_3_^2−^, C=O, and MgCO_3_ with merged curves (Figure 8). Moreover, curve fitting procedures based on the orbital pair component in Ca 2p3/2 and 2p1/2 are centered at 347.29 eV and 350 eV, respectively, corresponding to spin-orbital splitting belonging to calcium minerals. The binding energies of CO_3_^2−^ and C=O were found to be 531.77 eV and 533.60 eV, respectively, indicating that oxygen bonded with CaCO_3_ and molecular oxygen attached from the atmosphere [32,33]. CxHy and CO_3_^2−^ have binding energies of 284.60 eV and 289.28 eV, respectively. XPS analysis also shows that MgCO_3_ has a higher binding energy than the binding energy of any other nanoparticle’s individual elements, measured to be 1305 eV [34]. The carbon peaks show that the carbons attached with calcium, magnesium, and the reference carbon (adventitious) have decreasing BE values. The nanoparticle chemical environment can be explained by the start and peak spectra, and the ending peak also explained the FWHM (full width at half maximum) and atomic percentage [35]. The binding energy of C1s, Ca2p, Mg1s, and O1s has varying thickness, as described in Table 1.

## 4. Conclusions

The calcium and magnesium carbonates CaMg(CO_3_)_2_ that make up dolomite NPs are produced from dolomite limestone. They can be widely used because they are significant biological macro elements with potential techniques for mammalian/agricultural growth and development. For the production of CaMg(CO_3_)_2_ NPs, a ground mixture of dolomite limestone was synthesized by colloidal precipitates, and their features were investigated using XRD, particle size analysis by DLS, and surface morphology by SEM and TEM. The binding energy of each element of the dolomite nanoparticle was investigated using X-ray photoelectron spectroscopy. We investigated the applications of CaMg(CO_3_)_2_ NPs in seed germination, cancer cell growth inhibition, and antibacterial processes in this study. After employing 1% CaMg(CO_3_)_2_, seed germination was significantly improved. After utilizing 3% CaMg(CO_3_)_2_, we could reduce the human ovarian cancer cell line, SKOV3, whereas normal nHDH was unaffected. The bacteria growth was likewise observed to be greatly reduced in the CaCO_3_-treated group. In addition, the impacts of CaMg(CO_3_)_2_ NPs were investigated in a chicken in vivo investigation. The results revealed that the CaMg(CO_3_)_2_-NPs-treated group maintained a more uniform calcium absorption rate compared to the control group. According to our findings, CaMg(CO_3_)_2_ NPs function as a stimulant for plants and an inhibitory agent for bacteria and cancer cells.

## Figures and Tables

**Table 1:** SR-XPS C1s, Ca2p, Mg1s, O1s S2p core levels data collected on thick film of pure CaMg(CO_3_)_2_ nanoparticle stabilized by BE, FWHM, atomic ratio, and area.

Name	Start BE	Peak BE	End BE	Height CPS	FWHM eV	Area (P) CPS.eV	Area (N) TPP-2M	Atomic %
C1s	296	289.19	281	4906.63	2.15	25,060.28	352.4	35.1
Ca2p	355	347.3	342.03	16,307.06	2.21	57,068.56	139.64	13.91
Mg1s	1308.98	1304.97	1298	458.31	2.49	1283.33	4.35	0.43
O1s	540	531.81	526	29,711.11	2.73	87,514.01	507.65	50.56

## Data Availability

Data are available on request due to restrictions such as privacy or ethics.
